# StackPVP: a stacked ensemble classification framework for predicting phage virion proteins using integrated evolutionary features

**DOI:** 10.3389/fmicb.2026.1729937

**Published:** 2026-03-18

**Authors:** Lixi Cai, Huawei Zhang, Yunmeng Chu

**Affiliations:** 1 College of Basic Medicine, Putian University, Putian, Fujian, China; 2 Putian University Key Laboratory of Translational Tumor Medicine in Fujian Province, Putian, Fujian, China; 3 Center for AI-Driven Medical Research, Shenzhen Institutes of Advanced Technology, Chinese Academy of Sciences, Shenzhen, Guangdong, China; 4 Guangdong Provincial Key Laboratory of Nanhai Microbial Mineralization Technology and Application, Institute of Advanced Materials, Guangzhou Maritime University, Guangzhou, Guangdong, China

**Keywords:** evolutionary information, machine learning, phage virion protein, PSSM, stacked model

## Abstract

Phage therapy has attracted increasing attention as a potential antibacterial strategy. Phage virion proteins (PVPs) are vital for recognizing host cells and binding to their surface receptors. Thus, accurate PVP identification is essential for developing antibacterial agents. In this study, we introduced StackPVP, a computational approach that integrated ensemble learning methods with evolutionary features to improve PVP identification accuracy. Initially, position-specific scoring matrix (PSSM) were derived from protein sequences, from which additional features—including Amino Acid Composition derived from PSSM (AAC-PSSM), Dipeptide Composition derived from PSSM (DPC-PSSM), Pseudo Position-Specific Scoring Matrix (Pse-PSSM), and PSSM Composition (PSSM-COM)—were extracted. Subsequently, three feature selection methods were employed to determine the optimal feature subset, which was then used with 12 base machine learning classifiers. Among three meta-classifier algorithms—logistic regression (LR), random forest (RF), and support vector machine (SVM)—random forest achieved the best overall performance and was selected as the meta-classifier in the final stacking model. On the evaluated test dataset, StackPVP achieved an area under the curve (AUC) of 94.26% and showed improved specificity compared with several representative existing methods. These results suggest that StackPVP provides a complementary computational approach for PVP identification and may assist in phage genome annotation and related research.

## 1 Introduction

Bacteriophages, often referred as phages, are viruses that exclusively infect bacterial cells and serve a vital function in controlling microbial population dynamics (Dion et al., 2020). These viruses exhibit two life cycles: lysogenic and lytic. In the lysogenic cycle, phages integrate their genetic material into the host bacterium’s chromosome, establishing a stable coexistence with the host. In contrast, lytic phages utilize the host’s resources to replicate extensively within the cell, ultimately causing the host cell to lyse (Warwick-Dugdale et al., 2019; Dion et al., 2020). The recent increase in antibiotic-resistant bacterial strains represents a critical global health issue, underscoring the pressing necessity for the development of alternative antimicrobial approaches (Majumdar et al., 2022). Phage therapy, involving the use of bacteriophages to selectively target and eliminate bacterial pathogens, has gained recognition as a promising approach for combating multidrug-resistant organisms (Monteiro et al., 2019; Borin et al., 2021). The effectiveness of phage therapy relies greatly on accurately identifying the bacterial hosts of the phages (Borin et al., 2021; Federici et al., 2021). A comprehensive understanding of phage viral proteins (PVPs)—the structural proteins that compose the phage virion—is essential for elucidating phage-host interactions and facilitating the development of novel antibacterial interventions (Meng et al., 2020; Kabir et al., 2022). PVPs, consisting of structural parts like the capsid (head), tail, and related appendages, play essential roles in key functions such as recognizing the host, attaching to it, and injecting phage genetic material into bacterial cells. These proteins are of considerable interest due to their potential applications in innovative antibacterial therapeutics (Kabir et al., 2022). In contrast, non-virion proteins (non-PVPs) function intracellularly within the host to support processes such as viral replication and gene regulation but are not incorporated into the mature virion (Cao et al., 2024). Precise identification and functional analysis of PVPs are essential not only for enhancing the comprehension of virus-host interactions but also for optimizing the effectiveness of phage therapy and facilitating the development of novel antibacterial compounds (Roux et al., 2015; Guo et al., 2021). Despite their importance, identifying PVPs remains challenging due to the extensive diversity of protein sequences, limited availability of experimental data, and the multifaceted roles these proteins perform.

Techniques like mass spectrometry, protein arrays, and gel electrophoresis are well-established and dependable methods for identifying PVPs. However, these approaches are often constrained by high costs, labor-intensive procedures, and limited scalability. The emergence of high-throughput sequencing technologies has significantly expanded the accessibility of phage genomic data, thereby generating a need for computational approaches capable of efficiently and accurately predicting PVPs based on sequence information. To address this need, numerous machine learning (ML)-based approaches have been developed. Seguritan et al. (2012) created the first tool for predicting PVPs named iVIREONS, which utilized an artificial neural network (ANN) trained on characteristics like amino acid composition (AAC) and protein isoelectric point (PIP) to categorize phage proteins. Later, Feng et al. (2013) compiled a high-quality dataset consisting of 99 PVPs and 208 non-PVPs, referred to as the Feng2013 dataset, and developed a Naive Bayes (NB) prediction model based on AAC and dipeptide composition (DPC) features. In 2018, Manavalan et al. (2018) enhanced the dataset by merging the Feng2013 dataset with an additional set of 30 PVPs and 64 non-PVPs, resulting in the Manavalan2018 dataset. They also developed PVP-SVM, a classifier based on support vector machines (SVM) that utilizes features based on both composition and properties. Building upon this dataset, several advanced PVP prediction tools have been developed. For example, PVPred (Ding et al., 2014) employs an SVM classifier using g-gap dipeptide compositions, while PhagePred (Pan et al., 2018) utilizes a multinomial Naive Bayes classifier in conjunction with g-gap feature trees to characterize protein sequences. Tan et al. (2018) created an SVM predictor that integrates multiple optimal g-gap dipeptide composition sets. Pred-BVP-Unb (Arif et al., 2020) encodes proteins with three distinct feature types and applies an SVM classifier for PVP identification. PVPred-SCM (Charoenkwan et al., 2020b) represents proteins through dipeptide composition and uses a scoring card method classifier. The iPVP-MCV model (Han et al., 2021) was introduced as a multi-classifier voting system designed to improve predictive performance based on amino acid sequences. Phage_UniR_LGBM (Bao et al., 2022) combines UniRep features with the LightGBM algorithm for classification. Barman et al. (2023) explored both basic and ensemble machine learning techniques based on protein sequence composition features for PVP prediction. Moreover, Charoenkwan et al. (2020a) developed an additional widely adopted benchmark dataset, Charoenkwan2020_2.0, which facilitated the creation of Meta-iPVP (Charoenkwan et al., 2020a), a predictor that integrates seven types of protein features and employs four machine learning algorithms. This dataset has also been utilized in the development of other PVP predictors, such as SCORPION (Ahmad et al., 2022), which combines protein sequence features within a stacking-based ensemble framework, and PredPVP (Cao et al., 2024), an ensemble model that integrates various features in conjunction with feature selection methods to improve the precision of PVP prediction. Furthermore, Zhang et al. (2015) integrated four distinct categories of protein features and employed a random forest (RF) model to identify PVPs. PhANNs (Cantu et al., 2020) utilize a combination of diverse protein feature types to encode proteins and develop an artificial neural network (ANN) for classifying PVPs into specific subtypes. VirionFinder (Fang and Zhou, 2021) represents proteins using one-hot encoding combined with the biochemical properties of amino acids and applies a bi-path convolutional neural network (CNN) for PVP prediction. Although the aforementioned methods effectively facilitate the prediction of phage virion proteins (PVPs), several issues remain unresolved. First, due to the high sequence diversity of phage genomes, the conservation of protein amino acid sequences is relatively low (Chu et al., 2022). Consequently, conventional protein features may not adequately capture the distinctive characteristics of PVPs. To address this, we propose incorporating evolutionary information from the sequences to train the model. Additionally, previous studies have demonstrated that ensemble models can significantly enhance predictive performance (Ahmad et al., 2022; Chen et al., 2023; Cao et al., 2024); therefore, we employed a stacking method to train the model. Finally, the predictive accuracy of existing methods remains unsatisfactory for many practical therapeutic applications, highlighting the need for further improvements.

This study introduces StackPVP, a stacked ensemble learning framework for PVP identification using evolutionary information extracted from protein sequences. The method integrates multiple PSSM-derived descriptors and feature selection strategies, and combines diverse base classifiers with a meta-classifier to generate final predictions. Extensive cross-validation and independent evaluations indicate that StackPVP shows improved performance compared with several representative predictors under the evaluated datasets, and may support phage protein annotation and related research applications.

## 2 Materials and methods

### 2.1 Overview of StackPVP framework

**Figure 1:**
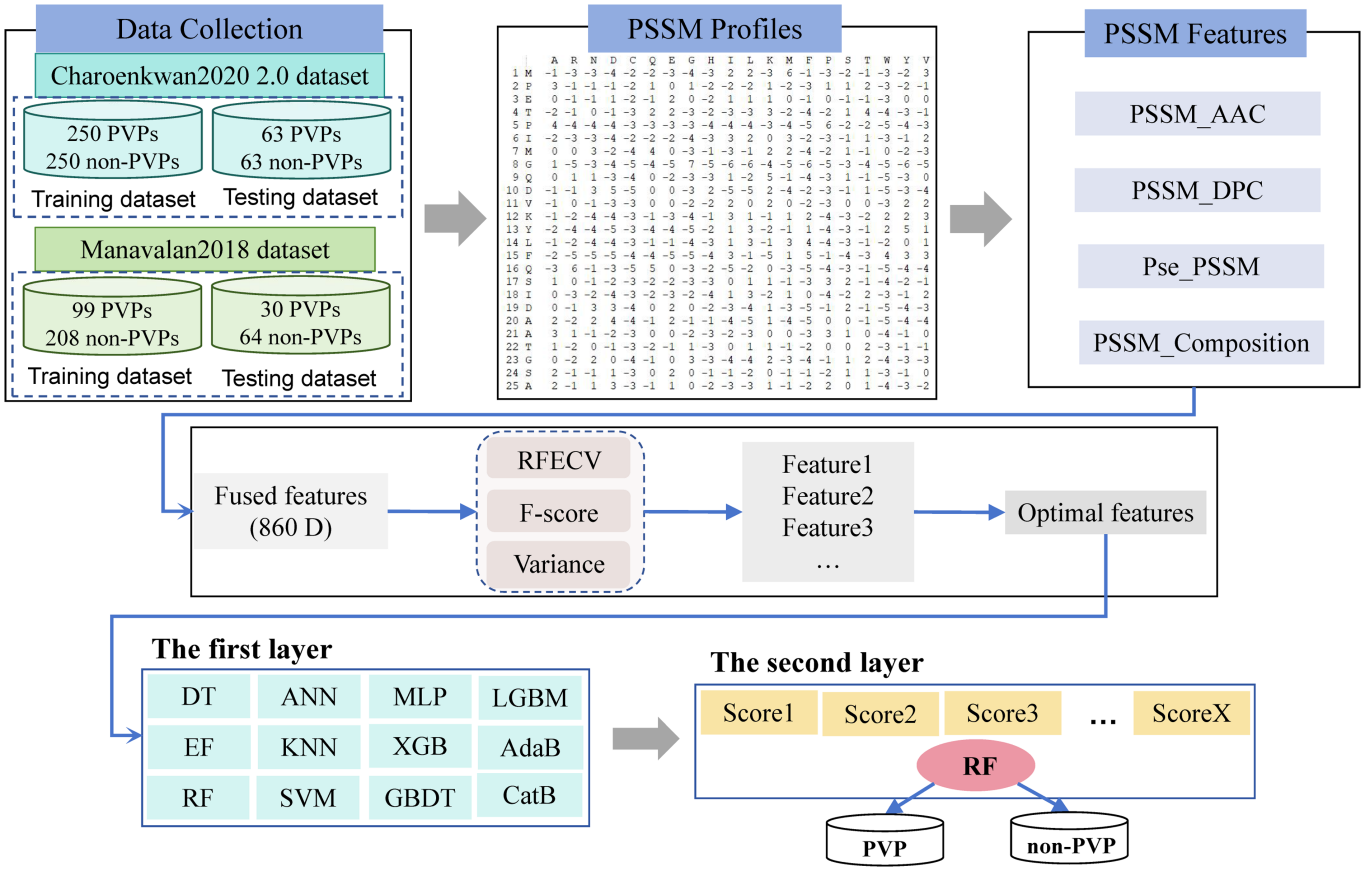
The flowchart of the StackPVP framework prediction model.

The methodological framework of this study, illustrated in Figure 1, comprises four primary phases: dataset construction, baseline model development, feature representation extraction, and the formulation of a stacked model. Initially, the benchmark dataset from Charoenkwan et al. (2020a) was used as the foundational resource for training and optimizing both the baseline models and the proposed StackPVP framework. Afterward, features based on the Position-Specific Scoring Matrix (PSSM) were extracted from the protein sequences. From these, additional feature sets were generated, including Amino Acid Composition derived from PSSM (AAC-PSSM), Dipeptide Composition derived from PSSM (DPC-PSSM), Pseudo Position-Specific Scoring Matrix (Pse-PSSM), and PSSM Composition (PSSM-COM), all based on the PSSM profiles. Next, dimensionality reduction and feature selection were performed using F-score, Variance, and Recursive Feature Elimination with Cross-Validation (RFECV) methods to determine the most informative set of features. Finally, a selection of base classifiers and meta-classifiers—including Logistic Regression (LR), RF, and SVM (Noble, 2006; van Smeden et al., 2019)—were used from a range of conventional machine learning algorithms to construct the final stacked ensemble model, StackPVP. The predictive performance of StackPVP was then evaluated and benchmarked against its individual baseline models as well as existing approaches.

### 2.2 Benchmark datasets

In this study, the Charoenkwan2020_2.0 dataset, meticulously curated by Charoenkwan et al. (2020a), was utilized to perform both cross-validation and independent testing of the proposed predictive model. This dataset is regarded as dependable for multiple reasons: (i) these proteins from the Charoenkwan2020_2.0 dataset were obtained from the UniProt database (release 2019_11) (Research at UC, 2019), and served as the benchmark dataset with the largest sample size currently available in PVP research; (ii) sequences containing non-standard amino acid residues such as X, U, Z, and B, as well as sequences shorter than 50 amino acids, were excluded to maintain data integrity; and (iii) the CD-HIT program (Fu et al., 2012) was employed to remove sequences exhibiting greater than 40% similarity, thereby minimizing redundancy. The final dataset consisted of 313 PVPs and 313 non-PVPs. The training and independent datasets contained 250 PVPs/250 non-PVPs and 63 PVPs/63 non-PVPs, respectively, following previous reports. To further assess the robustness and generalizability of the proposed model, the widely recognized Manavalan2018 dataset (Manavalan et al., 2018) was also used.

### 2.3 Extraction of features

Feature extraction is essential in protein identification, as it transforms a protein’s amino acid sequence into unique, fixed-length feature vectors. This process enables effective characterization of proteins through attributes derived from the encoded sequence data. Earlier research has shown that combining the physicochemical characteristics and evolutionary data of amino acids offers a more complete depiction of protein attributes. In this context, PSSM are first generated and then used to derive multiple feature sets, including AAC-PSSM, DPC-PSSM, Pse-PSSM, and PSSM-COM. These four PSSM-derived descriptors were selected because they capture complementary evolutionary characteristics, including global composition (AAC-PSSM), local residue-pair dependency (DPC-PSSM), sequence-order correlation (Pse-PSSM), and position-aware compositional patterns (PSSM-COM). These features were chosen because they have been reported to be effective in capturing conserved motifs that are important for the structural integrity of phage virions. These feature sets effectively capture the evolutionary information inherent in protein sequences, thereby enhancing classification performance. Comprehensive explanations of these feature extraction techniques are given in the subsections below.

The PSSM is an L × 20 matrix, where L is the length of the protein sequence and 20 represents the standard amino acids. Each element at position (i, j), with 1 ≤ i ≤ L and 1 ≤ j ≤ 20, indicates the log-odds substitution score of the j-th amino acid at position i. To construct the PSSM, PSI-BLAST version 2.2.26 was used to query the protein sequence against the UniRef50 database,^1^ employing an E-value cutoff of 0.001 and performing three iterative search cycles (Thung et al., 2021). Rather than deriving features directly from primary protein sequences, evolutionary features offer deeper insights by leveraging profile information from the PSSM. Using the POSSUM toolkit (Wang et al., 2017), four categories of evolutionary features were extracted: AAC-PSSM (Liu et al., 2010), DPC-PSSM (Liu et al., 2010), PSSM-COM (Zou et al., 2013), and Pse-PSSM (Chou and Shen, 2007). The computational methods underlying these PSSM-based features are briefly outlined below.

The AAC-PSSM feature vector is derived by averaging the value of each amino acid at every sequence position, as illustrated in the Equation (1):


$$x_{j}=\frac{1}{L}\sum_{k=1}^{L-1}p_{i,j}\;(j=1,2,\ldots ,20),$$ (1)

where x_j_ (1 ≤ j ≤ 20) represents the composition of the j-th amino acid type in the PSSM, indicating the average score of amino acid residues in protein S mutated to the j-th type during evolution.

The DPC-PSSM is derived by computing the sum and average of the products of the ith and jth amino acids across two successive rows of the PSSM, as expressed in the Equation (2):


$$y_{i,j}=\frac{1}{L-1}\sum_{k=1}^{L-1}P_{k,j}\times p_{k+1,j}\;(1\leq i,j\leq 20).$$ (2)

The PSSM-COM, derived from the original PSSM row transformation, is a 20 × 20 matrix calculated as the Equations (3) and (4):


$$R_{i}=\sum_{k=1}^{N}r_{k}\times \delta_{k},$$ (3)

where


$$\left\{ \begin{array}{c} \delta_{k}=1,\text{when}\;p_{k}=a_{i}\\ \delta_{k}=0,\text{when}\;p_{k}\neq a_{i} \end{array}\;(i=1,2,\ldots ,20),\right.$$ (4)

$R_{i}$ is the *i*th row of the newly generated matrix, $r_{k}$ is the *k*th row of the PSSM matrix, *k* ranges from 1 to *N*, *N* is the length of the sequence, $p_{k}$ denotes the amino acid at the *k*th position in the sequence and $a_{i}$ represents the *i*th standard amino acid.

The Pse-PSSM, based on the concept of the PseAAC encoding algorithm applied to PSSM, the Pse-PSSM method has been introduced to capture both the overall amino acid sequence information and the local sequence-order effects (Chou and Shen, 2007; Yang et al., 2023). It is defined as the Equation (5)


$$D_{s}=\frac{1}{L}\sum_{i=1}^{L}{\left(p_{i,s}-{\bar{p}}_{s}\right)}^{2\;\;\;}({\bar{p}}_{s}=\sum_{i=1}^{L}p_{i,s},s=1,2,\ldots ,10;i=1,2,\ldots ,L),$$ (5)

where *p*_*i*,s_ represents the pseudo-composition of the amino acid a*_i_* when it is mutated to “*s*.”

Additionally, five widely used feature descriptors were employed for comparative evaluation: AAC, DPC, GAAC (group amino acid composition), GDPC (group dipeptide composition), and the composition (CTDC), transition (CTDT), and distribution (CTDD) components derived from the composition-transition-distribution (CTD) framework. Specifically, AAC and DPC quantify the occurrence frequencies of single amino acid residues and pairs of amino acids (dipeptides) in protein sequences, respectively. GAAC and GDPC capture properties that represent the physicochemical characteristics of grouped amino acids. These sequence-based descriptors combine composition, composition-transition-distribution measures, position-specific information, and physicochemical characteristics to create a strong predictive model. The computation of these feature descriptors was performed using the iFeature Python package (Chen et al., 2018).

### 2.4 Methods for selecting features

Selecting the right features is a crucial part of the feature engineering process (Armanfard et al., 2016). This process, akin to dimensionality reduction, aims to eliminate redundant or irrelevant features, thereby reducing dimensionality and enhancing the computational efficiency of machine learning models. The primary strategies for feature selection include filter, wrapper, and embedded methods. Notably, filter and wrapper techniques are generally considered more effective for feature selection than embedded methods (Jiang et al., 2021). In this study, feature selection is performed using filter-based approaches—specifically, the F-score and Variance methods—along with a wrapper-based technique, RFECV (Li et al., 2021).

The F-score method (Song Q. et al., 2017) is derived from the F-test, a statistical procedure commonly used in hypothesis testing, particularly within the framework of Analysis of Variance (ANOVA). Its main objective is to evaluate the linear correlation between each individual feature and the target variable. The primary null hypothesis asserts that no statistically significant linear relationship exists between the specified feature and the outcome variable. The calculated *F*-value serves as a metric to determine whether this null hypothesis can be rejected, thereby enabling the selection of features that exhibit a strong correlation with the target. The Variance Selection method (Henseler et al., 2015) eliminates features exhibiting variance below a specified threshold, as these features are deemed less informative for differentiating among samples. RFECV (Li et al., 2021) is a greedy optimization method that iteratively builds models by retaining the most relevant features at each step. In subsequent iterations, the algorithm constructs models using features not previously retained, continuing this process until all features have been evaluated. Ultimately, features are ranked based on the order in which they were retained, facilitating the identification of an optimal subset of features.

### 2.5 Machine learning algorithms

#### 2.5.1 Stacking ensemble model

Studies have demonstrated that utilizing a stacked ensemble learning approach can improve the predictive accuracy across a range of bioinformatics applications (Xiong et al., 2018; Zhang et al., 2021). Distinct from other ensemble learning methods, this approach allows for the seamless integration of multiple machine learning classifiers to build a unified, effective predictive model. The stacking approach involves two primary stages, where the models used in each stage are called the base models and the meta models, respectively. Herein, we constructed a two-layer stacked ensemble classifier (Naimi and Balzer, 2018), and the framework of this classifier is illustrated in Figure 1. Features of PVPs and non-PVPs were extracted using evolutionary information. In the first layer, after feature dimensionality reduction, the training dataset was used to train the base models with 10-fold cross-validation, generating predicted values. In the second layer, the outputs from the base models were merged and input into the second-layer classifiers to generate the final predictions. During training, model fitting followed the standard optimization procedures of the corresponding algorithms; for models requiring iterative optimization (e.g., LR/ANN/MLP), parameters were learned using gradient-based methods, and the final settings were selected based on cross-validation performance to support reproducibility.

#### 2.5.2 Classification algorithm

Decision Tree (DT) is a supervised machine learning method used for classification and regression (Song and Lu, 2015). It resembles a tree, with branches representing decision rules, internal nodes as dataset features, and leaf nodes as outcomes.

Random Forest (RF) is an ensemble learning method that improves predictive accuracy and model stability by combining multiple decision trees (Biau, 2012). Each tree is built from random subsets of training data, and the final prediction aggregates their outputs. This approach enhances generalization and robustness, reducing overfitting risk.

Extreme Random Tree (EF) resemble RF but incorporate greater randomness (Geurts et al., 2006). EF is an ensemble learning method that randomizes both data sample selection and feature split points during tree construction. This approach boosts model diversity, reduces variance, and often improves generalization and robustness.

Gradient Boosting Decision Tree (GBDT) is a powerful machine learning method that sequentially builds an ensemble of decision trees, with each tree correcting errors from the previous ones (Zhang et al., 2019). This iterative process produces a highly accurate and generalizable predictive model.

Extreme Gradient Boosting (XGBoost) is a highly optimized, scalable variant of gradient boosting designed for computational efficiency and superior performance (Chen, 2016). It enhances the traditional GBDT by adding regularization, parallel processing, and advanced tree pruning. XGBoost is widely used for structured data tasks, employing second-order Taylor expansions of the loss function to improve accuracy and regularization to control model complexity.

Light Gradient Boosting Machine (LightGBM) is an advanced extension of the GBDT framework, widely used for classification and prediction (Ke et al., 2017). Building on XGBoost, LightGBM incorporates novel techniques like Gradient-based One-Side Sampling (GOSS), Exclusive Feature Bundling (EFB), and a histogram-based approach. These innovations reduce memory usage and improve training efficiency while maintaining predictive accuracy comparable to XGBoost.

Adaptive Boosting (AdaBoost) is a pioneering and influential boosting algorithm that combines multiple weak learners, typically shallow decision trees, to build a highly accurate and robust model. During training, AdaBoost iteratively adjusts the weights of misclassified instances, focusing subsequent learners on harder cases (Wang, 2012).

Categorical Boosting (CatBoost) is an advanced gradient boosting algorithm that efficiently handles categorical variables (Hancock and Khoshgoftaar, 2020). Unlike traditional methods, CatBoost automatically processes categorical features, minimizing preprocessing, reducing information loss, and enhancing model robustness. It employs ordered boosting and permutation-based training to reduce overfitting and improve generalization, especially in datasets with diverse feature types.

Support Vector Machine (SVM) is a robust supervised learning algorithm for classification and regression (Noble, 2006). It identifies the optimal hyperplane that maximizes the margin between classes in feature space, improving performance in high-dimensional settings. For nonlinear data, SVM employs kernel functions to enhance class separation.

Artificial Neural Network (ANN), also known as a feedforward neural network, consists of an input layer, one or more hidden layers, and an output layer (Abiodun et al., 2018). The number of hidden layers and neurons significantly influences the model’s performance.

K-Nearest Neighbors (KNN) is one of the simplest classification algorithms (Song Y. S. et al., 2017). It classifies unknown samples based on their nearest k neighbors. Therefore, selecting an appropriate value for k is crucial: if k is too large, the model tends to underfit; if k is too small, it may overfit. The KNN algorithm is widely used in classification due to its simplicity and ease of implementation (Abu Alfeilat et al., 2019).

Multi-Layer Perceptron (MLP) is a feedforward artificial neural network comprising an input layer, one or more hidden layers, and an output layer (Tang et al., 2015). It models complex nonlinear relationships by using backpropagation and gradient descent. Unlike linear models, the MLP utilizes multiple hidden layers and nonlinear activation functions to create hierarchical representations, allowing it to capture intricate patterns in high-dimensional data. Iterative parameter optimization via backpropagation and gradient descent enhances its capacity to approximate highly nonlinear functions.

### 2.6 Performance evaluation

This study employs standard evaluation metrics: accuracy (ACC), sensitivity (Sn), specificity (Sp), F1-score (F1), Precision, and Matthews correlation coefficient (MCC). Classifier performance is further evaluated via the receiver operating characteristic (ROC) curve and its area under the curve (AUC) (Li, 2024). Definitions of these metrics are as follows:


$$\mathrm{ACC}=\frac{\mathrm{TP}+\mathrm{TN}}{\mathrm{TP}+\mathrm{TN}+\mathrm{FP}+\mathrm{FN}}$$

$$\mathrm{Sn}=\frac{\mathrm{TP}}{\mathrm{TP}+\mathrm{FN}}$$

$$\mathrm{Sp}=\frac{\mathrm{TN}}{\mathrm{TN}+\mathrm{FP}}$$

$$\text{Precision}=\frac{\mathrm{TP}}{\mathrm{TP}+\mathrm{FP}}$$

$$\mathrm{MCC}=\frac{\mathrm{TP}\times \mathrm{TN}-\mathrm{FP}\times \mathrm{FN}}{\sqrt{\left(\mathrm{TP}+\mathrm{FN})(\mathrm{TP}+\mathrm{FP})(\mathrm{TN}+\mathrm{FP})(\mathrm{TN}+\mathrm{FN}\right)}}$$

$$F1=2\times \frac{\mathrm{TP}}{2\mathrm{TP}+\mathrm{FP}+\mathrm{FN}}$$

$$\mathrm{AUC}=\frac{1}{2}(\frac{\mathrm{TP}}{\mathrm{TP}+\mathrm{FN}}+\frac{\mathrm{TN}}{\mathrm{TN}+\mathrm{FP}})$$

TP, TN, FP, and FN represent true positive, true negative, false positive, and false negative rates, respectively. The AUC ranges from 0 to 1, with values near 1 indicating better model performance. This study used 10-fold cross-validation on the training set and calculated the AUC of the ROC curve for all cases.

## 3 Results and discussion

### 3.1 Evaluation of different feature extraction methods

**Figure 2:**
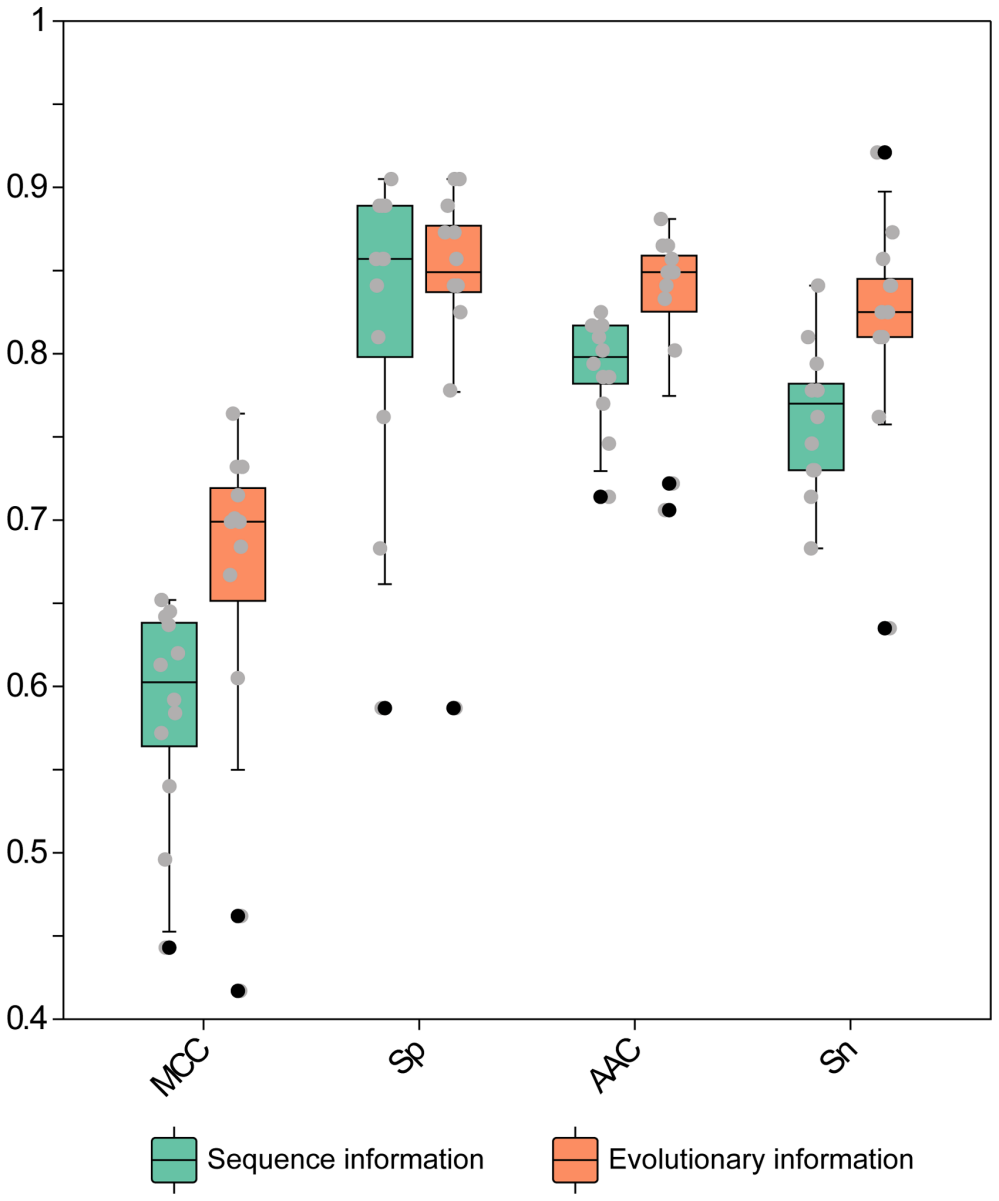
Performance evaluation of 12 machine learning classifiers using various feature extraction methods on the testing dataset. Each point represents the evaluation metric of an individual base classifier.

**Figure 3:**
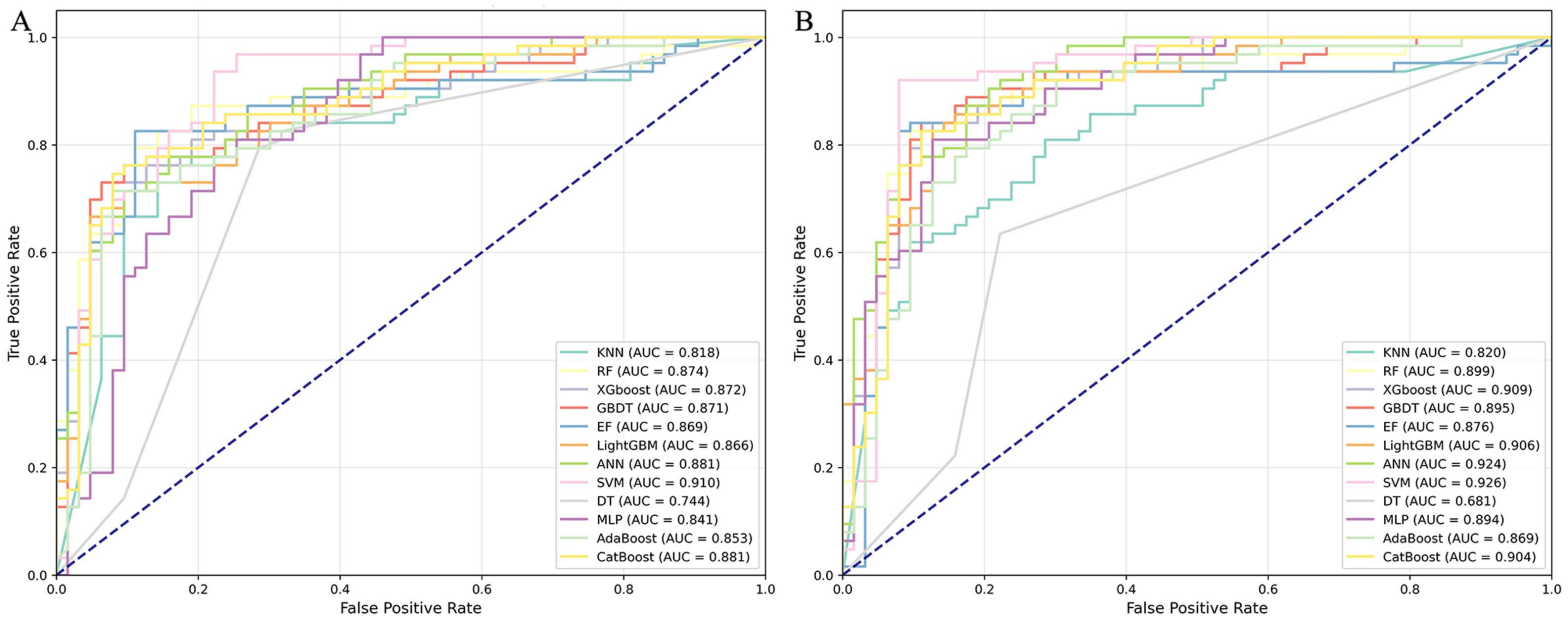
ROC curves of the base classifiers on the testing dataset: **(A)** sequence information; **(B)** evolutionary information.

Recognizing the importance of both physical and evolutionary attributes, we selected 723 commonly used amino acid sequences and physical characteristics—including AAC, DPC, and CTD—to compare with 860 sequence evolutionary features (AAC-PSSM, DPC-PSSM, Pse-PSSM, and PSSM-COM). We employed 12 machine learning algorithms, training models separately on all evolutionary information and all sequence information. We then evaluated the models using the test dataset, with the results shown in Figure 2; Supplementary Table S1. Overall, 12 machine learning classifiers using all evolutionary features achieved improved performance, with average increases in AAC, Sp, Sn, Precision, F1 score, MCC, and AUC of 3.64, 1.59, 5.69, 1.95, 4.03, 7.01, and 1.86%, respectively, compared to models trained on all sequence information. Among these evaluation metrics, models trained on evolutionary features achieved the highest values: AAC of 0.8810 (SVM), Sp of 0.9048 (RF and EF), Sn of 0.9206 (SVM), Precision of 0.8966 (RF and EF), F1 score of 0.8855 (SVM), and MCC of 0.7643 (SVM). In contrast, models trained on sequence information features achieved their highest values as follows: AAC of 0.8254 (SVM), Sp of 0.9048 (XGBoost), Sn of 0.8413 (KNN), Precision of 0.8846 (XGBoost), F1 of 0.8197 (SVM), and MCC of 0.6521 (SVM). Except for Sp, the evaluation metrics obtained from models trained with evolutionary features outperformed those from sequence information features. Furthermore, we assessed the classifier’s performance on the testing set using the ROC curve and AUC metrics. As illustrated in Figure 3; Supplementary Table S1, the average AUC values for models trained on all sequence information were 0.8185 (KNN), 0.8738 (RF), 0.8718 (XGBoost), 0.8707 (GBDT), 0.8690 (EF), 0.8662 (LightGBM), 0.8808 (ANN), 0.9098 (SVM), 0.7444 (DT), 0.8408 (MLP), 0.8535 (AdaBoost), and 0.8806 (CatBoost). Compared to models trained on sequence information, the corresponding base classifiers trained on evolutionary features performed better, with AUC improvements of 0.14% (KNN), 0.26% (RF), 3.75% (XGBoost), 2.47% (GBDT), 0.66% (EF), 3.96% (LightGBM), 4.36% (ANN), 1.66% (SVM), 5.37% (MLP), 1.52% (AdaBoost), and 2.29% (CatBoost). In conclusion, the 12 classification algorithms performed better on all evolutionary features than on all features. Therefore, given the consistent empirical advantage observed under the evaluated setting, we selected the evolutionary-feature set to develop the final stacked model in this study.

### 3.2 Evaluation of various dimensionality reduction methods

**Figure 4:**
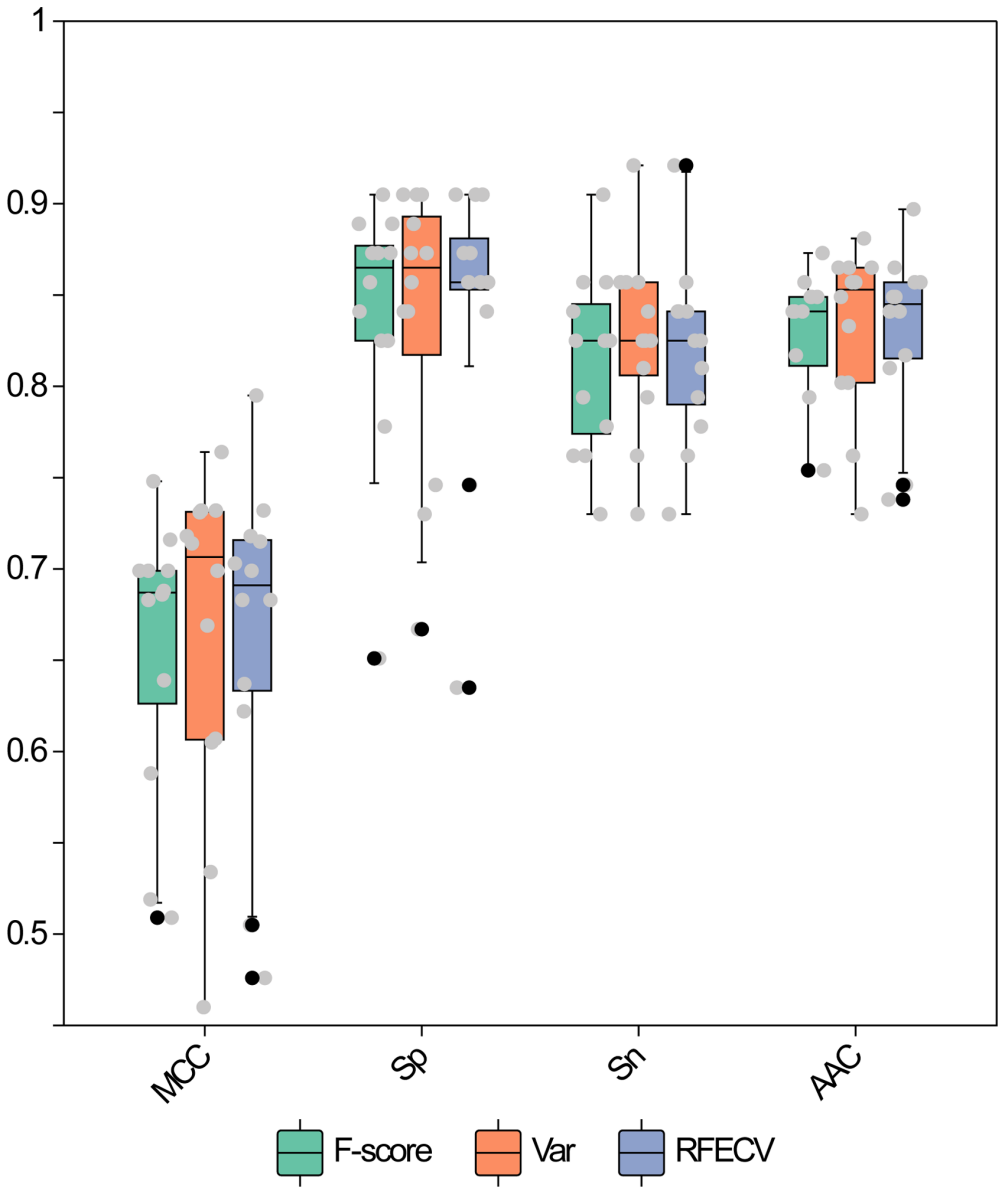
Performance evaluation of base classifiers using various feature dimensionality reduction methods on the testing dataset. Each point represents the evaluation metric of an individual base classifier.

**Figure 5:**
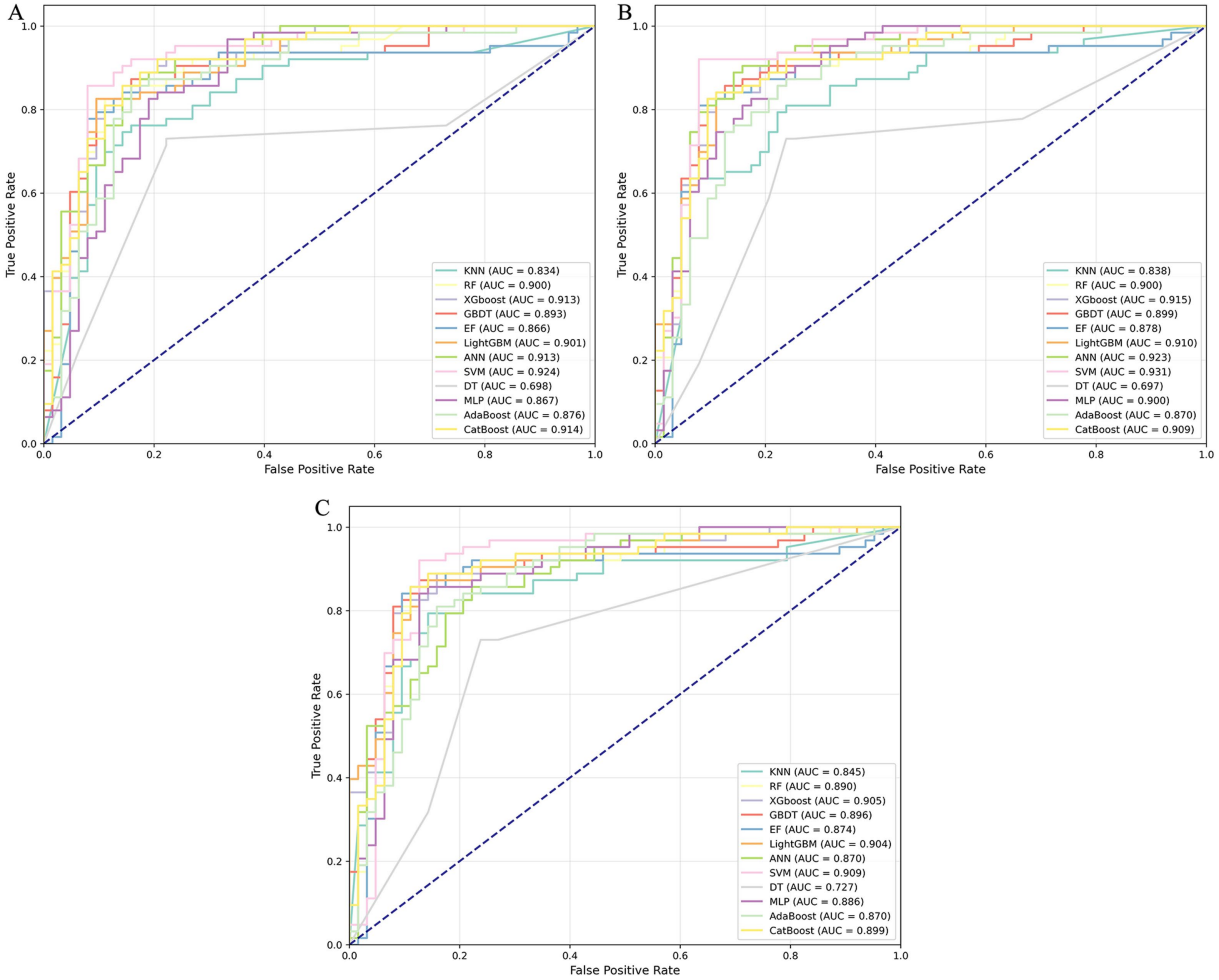
The ROC curves of the basic classifiers, trained using features obtained through different dimensionality reduction methods on the test dataset, are shown: **(A)** F-score, **(B)** RFECV, and **(C)** Variance.

In the course of the machine learning process, the presence of high-dimensional input feature vectors can lead to increased model complexity and a consequent decline in the model’s generalization capability. Therefore, it is necessary to reduce feature dimensionality to enhance model performance. Three algorithms—F-score, Variance, and RFECV—were utilized to perform dimensionality reduction on the evolutionary features. Initially, we performed 10-fold cross-validation on each of the three feature datasets employing 12 base algorithms, which led to the training of a total of 36 base models. Subsequently, the performance of these models was assessed using the test dataset. The evaluation performance of the 12 base classifiers on datasets with different dimensions of evolutionary features is presented in Figure 4; Supplementary Table S2. Although the 12 classification algorithms yielded varying values for AAC, Sp, Sn, and MCC on the same reduced-dimensional dataset, overall model performance across different reduced-dimensional datasets did not show significant differences. Furthermore, after dimensionality reduction of all evolutionary features, the performance of the base models remained largely unchanged or even improved. For example, the average AAC, Sp, MCC, and F1 values for Variance (0.8307, 0.8360, 0.6638, 0.8303) and RFECV (0.8307, 0.8426, 0.6638, 0.8291) were slightly higher than those for all features (0.8267, 0.8346, 0.6564, 0.8253). The Sn value for Variance (0.8254) was higher than that for all features (0.8188), and the average Precision values for F-score (0.8389), Variance (0.8383), and RFECV (0.8425) exceeded the average Precision of the full-feature models (0.8357). Additionally, the AUC values were compared and are shown in Figure 5; Supplementary Table S2. Among the 12 models using Variance, only three had an AUC value exceeding 0.9, whereas F-score and RFECV had six and seven models, with AUC values above 0.9, respectively. Notably, among the SVM models in the RFECV dataset, the AUC reached as high as 0.9307, making it the best-performing model among all 36 base models.

### 3.3 Performance of various meta-classifier models

**Figure 6:**
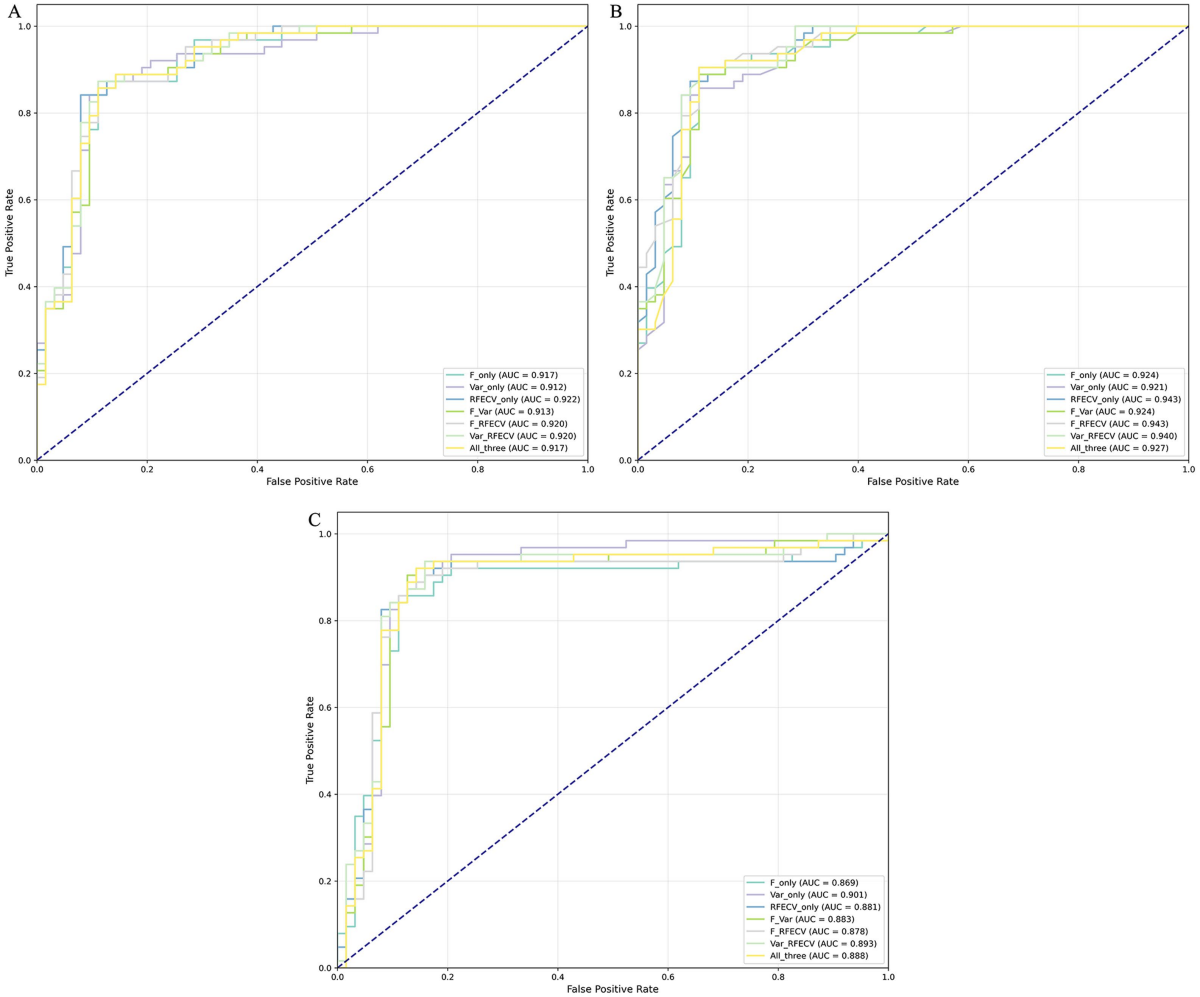
The ROC curves of various meta-classifier models on the test dataset: **(A)** LR, **(B)** RF, and **(C)** SVM.

Given that these fundamental classifiers demonstrate minimal variability across reduced-dimensional feature sets, we use each base classifier’s outputs as new protein feature representations. These features are then fed into the meta-classifier to train an optimal predictive model. Selecting the meta-classifier is crucial for developing the stacked model. This study evaluated three machine learning algorithms—LR, RF, and SVM—as meta-classifiers using reduced-dimensional feature datasets, resulting in 21 stacked ensemble classifiers. Ten-fold cross-validation results on the training set are shown in Supplementary Table S3, with testing performance metrics in Figure 6; Table 1. The performance of the ensemble model utilizing various meta-classifiers was assessed through the ROC curve across multiple datasets and their combinations. When the RF algorithm served as the meta-classifier, the ROC curve exhibited the highest AUC on the independent testing set. Specifically, the AUC values for RF as the meta-classifier were 99.56 and 99.41% during 10-fold cross-validation (Supplementary Table S3), and 94.29 and 94.26% when evaluated on the RFECV and F_RFECV testing sets (Table 1), respectively. Furthermore, the ensemble model achieves the best performance on the testing set when RF is used as the meta-classifier, attaining the highest ACC, Sn, MCC, F1, and AUC values of 0.8968, 0.9048, 0.7938, 0.8976, and 0.9426, respectively. The highest Sp and Precision were observed when LR was applied to the RFECV dataset, reaching 0.9206 and 0.9118, respectively. This model also demonstrated strong performance in other metrics, including an ACC of 0.8730, AUC of 0.9221, Sn of 0.8254, MCC of 0.7494, and F1 of 0.8667. Compared to the RF meta-classifier applied to the RFECV dataset, there were slight differences in performance: ACC was 0.8810, AUC 0.9429, Sn 0.8889, MCC 0.7620, and F1 0.8819. Considering all metrics collectively, we conclude that the model using RF as the meta-classifier outperforms the model using LR. Furthermore, when RF is used as the meta-classifier, models based on the F_RFECV dataset achieved ACC, Sn, MCC, and F1 values of 0.8968, 0.9048, 0.7938, and 0.8976, respectively. These indicators represent the best performance among all models, with the AUC value also nearly the highest—0.9426 for the F_RFECV model compared to 0.9429 for the RFECV model. In summary, considering all evaluation metrics, the ensemble model built with the F_RFECV dataset and utilizing RF as the meta-classifier exhibits the best performance. Consequently, we developed a two-layer stacked model comprising 12 machine learning algorithms in the first layer, with RF serving as the meta-classifier, to effectively identify PVPs.

**Table 1:** Performance of different meta-classifier models on the test dataset.

Meta-classifier	Dataset	ACC	Sn	Sp	Precision	F1	MCC	AUC
LR	F	0.8492	0.8095	0.8889	0.8793	0.8430	0.7006	0.9166
	Var	0.8651	0.8254	0.9048	0.8966	0.8595	0.7325	0.9118
	RFECV	0.8730	0.8254	0.9206	0.9123	0.8667	0.7494	0.9221
	F_Var	0.8492	0.8095	0.8889	0.8793	0.8430	0.7006	0.9128
	F_RFECV	0.8571	0.8254	0.8889	0.8814	0.8525	0.7157	0.9199
	Var_RFECV	0.8571	0.8095	0.9048	0.8947	0.8500	0.7175	0.9196
	All_three	0.8492	0.8095	0.8889	0.8793	0.8430	0.7006	0.9174
RF	F	0.8492	0.8095	0.8889	0.8793	0.8430	0.7006	0.9243
	Var	0.8571	0.8095	0.9048	0.8947	0.8500	0.7175	0.9214
	RFECV	0.8810	0.8889	0.8730	0.8750	0.8819	0.7620	0.9429
	F_Var	0.8651	0.8413	0.8889	0.8833	0.8618	0.7310	0.9243
	F_RFECV	0.8968	0.9048	0.8889	0.8906	0.8976	0.7938	0.9426
	Var_RFECV	0.8810	0.8571	0.9048	0.9000	0.8780	0.7628	0.9397
	All_three	0.8730	0.8571	0.8889	0.8852	0.8710	0.7464	0.9273
SVM	F	0.8571	0.8254	0.8889	0.8814	0.8525	0.7157	0.8690
	Var	0.8571	0.8413	0.8730	0.8689	0.8548	0.7146	0.9010
	RFECV	0.8730	0.8413	0.9048	0.8983	0.8689	0.7475	0.8811
	F_Var	0.8571	0.8254	0.8889	0.8814	0.8525	0.7157	0.8826
	F_RFECV	0.8730	0.8571	0.8889	0.8852	0.8710	0.7464	0.8783
	Var_RFECV	0.8651	0.8413	0.8889	0.8833	0.8618	0.7310	0.8929
	All_three	0.8651	0.8413	0.8889	0.8833	0.8618	0.7310	0.8876

### 3.4 Comparison of StackPVP with previous methods

Using the methodology described above, we developed StackPVP and evaluated it under the same dataset setting to reduce data-related confounding factors. All comparative results are based on the same benchmark dataset and evaluation protocol reported in the original publications, to ensure fair comparison. Specifically, we compared StackPVP with existing predictors using the Charoenkwan2020_2.0 benchmark and the identical independent test set reported in prior work. As shown in Table 2, StackPVP achieved competitive performance and showed higher AUC under this benchmark (AUC = 94.26%). We present this result as an observed improvement under the evaluated experimental setting, while noting that further ablation analyses would be needed to fully isolate the contribution of individual pipeline components. Therefore, StackPVP provides an additional computational approach for PVP identification under the evaluated benchmark setting.

**Table 2:** Results of StackPVP and existing predictors on the Charoenkwan2020_2.0 dataset.

Methods^a^	ACC	Sn	Sp	MCC	AUC
PVPred	0.730	0.892	0.663	0.505	0.857
PVP-SVM	0.746	0.816	0.701	0.505	0.844
iPVP-MCV	0.833	0.889	0.778	0.671	0.883
SCORPION	0.881	0.810	0.952	0.770	0.922
PredPVP	0.897	0.921	0.873	0.795	0.934
StackPVP	0.8968	0.9047	0.8888	0.7937	0.9426

^a^Results of existing predictors from PredPVP (Cao et al., 2024).

To further validate the robustness of the StackPVP predictor, we employed an additional benchmark dataset constructed by Manavalan et al. (2018) and compared StackPVP with existing methods that used the same dataset. As shown in Table 3, StackPVP achieved comparable results on this imbalanced dataset to those obtained with the balanced dataset, demonstrating improved performance under the same evaluation protocol over existing models in evaluation metrics such as ACC, AUC, MCC, Sn, and Sp. Specifically, compared to other models, our method improved ACC by 4.29%, Sn by 16.63%, MCC by 11.65%, and AUC by 9.21%. These results suggest that StackPVP remains effective on this widely used imbalanced benchmark under the evaluated protocol; however, this benchmark-based evaluation does not fully guarantee generalization to future or phylogenetically distant PVPs.

**Table 3:** Results of StackPVP and other existing predictors on the Manavalan2018 dataset.

Methods^a^	ACC	Sn	Sp	MCC	AUC
PVPred	0.713	0.600	0.765	0.357	0.742
PVP-SVM	0.798	0.667	0.859	0.531	0.844
Tan et al’s method	0.755	0.700	0.781	0.464	0.651
PVPred-SCM	0.777	0.767	0.781	0.523	0.781
iPVP-MCV	0.840	0.667	0.922	0.621	0.820
PredPVP	0.872	0.700	**0.953**	0.698	0.873
StackPVP	0.9149	0.9333	0.9062	0.8145	0.9651

^a^Results of existing predictors from PredPVP (Cao et al., 2024).

## 4 Conclusion

In this study, we developed StackPVP, a stacked ensemble framework for phage virion protein identification using PSSM-derived evolutionary descriptors (AAC-PSSM, DPC-PSSM, Pse-PSSM, and PSSM-COM) together with feature selection strategies (F-score, Variance, and RFECV). By integrating outputs from multiple base classifiers with a random forest meta-classifier, StackPVP achieved an AUC of 0.9426 on the evaluated independent test set and showed an empirical improvement compared with several representative predictors under the same benchmark setting.

At the same time, several limitations should be acknowledged. First, because PSSM profiles rely on detectable homologs, the method may be less effective for orphan or highly novel proteins where PSI-BLAST produces sparse or uninformative profiles. Second, PSSM-based features mainly reflect local evolutionary conservation and do not explicitly capture long-range interactions or higher-order structural motifs, which may also contribute to virion protein function. Third, the observed performance gains are modest, and given the limited size and diversity of currently available benchmark datasets, increasingly complex ensemble strategies may yield diminishing returns under the current data scale and feature paradigm.

In addition, generating PSSM profiles introduces additional computational overhead compared with purely sequence-derived features. Since we did not perform a dedicated runtime benchmarking analysis across tools in this work, StackPVP should be viewed as an accuracy-oriented approach that may be most suitable for focused annotation and candidate refinement rather than high-throughput screening under strict computational constraints. Future work will benefit from more diverse datasets and from integrating complementary representations (e.g., structure-aware features or representation learning) to further improve robustness and practical applicability.

## Data Availability

The original contributions presented in the study are included in the article/Supplementary material. The data and codes used in this study are available at https://github.com/virbioinfor/StackPVP_2025. Further inquiries can be directed to the corresponding authors.

## Footnotes

1
https://www.uniprot.org/help/uniref

## References

[ref1] Abiodun O. I. Jantan A. Omolara A. E. Dada K. V. Mohamed N. A. Arshad H. (2018). State-of-the-art in artificial neural network applications: a survey. Heliyon 4:e00938. doi: 10.1016/j.heliyon.2018.e00938, 30519653 PMC6260436

[ref2] Abu Alfeilat H. A. Hassanat A. B. A. Lasassmeh O. Tarawneh A. S. Alhasanat M. B. Eyal Salman H. S. . (2019). Effects of distance measure choice on K-nearest neighbor classifier performance: a review. Big Data 7, 221–248. doi: 10.1089/big.2018.0175 31411491

[ref3] Ahmad S. Charoenkwan P. Quinn J. M. W. Moni M. A. Hasan M. M. Lio P. . (2022). SCORPION is a stacking-based ensemble learning framework for accurate prediction of phage virion proteins. Sci. Rep. 12:4106. doi: 10.1038/s41598-022-08173-5, 35260777 PMC8904530

[ref4] Arif M. Ali F. Ahmad S. Kabir M. Ali Z. Hayat M. (2020). Pred-BVP-Unb: fast prediction of bacteriophage virion proteins using un-biased multi-perspective properties with recursive feature elimination. Genomics 112, 1565–1574. doi: 10.1016/j.ygeno.2019.09.006, 31526842

[ref5] Armanfard N. Reilly J. P. Komeili M. (2016). Local feature selection for data classification. IEEE Trans. Pattern Anal. Mach. Intell. 38, 1217–1227. doi: 10.1109/TPAMI.2015.2478471, 26390448

[ref6] Bao W. Cui Q. Chen B. Yang B. (2022). Phage_UniR_LGBM: phage virion proteins classification with UniRep features and LightGBM model. Comput. Math. Methods Med. 2022:9470683. doi: 10.1155/2022/9470683, 35465015 PMC9033350

[ref7] Barman R. K. Chakrabarti A. K. Dutta S. (2023). Prediction of phage virion proteins using machine learning methods. Molecules 28:2238. doi: 10.3390/molecules28052238, 36903484 PMC10004995

[ref8] Biau G. (2012) Analysis of a random forests model. J. Mach. Learn. Res. 13: 1063–1095. doi: abs/10.5555/2503308.2343682.

[ref9] Borin J. M. Avrani S. Barrick J. E. Petrie K. L. Meyer J. R. (2021). Coevolutionary phage training leads to greater bacterial suppression and delays the evolution of phage resistance. Proc. Natl. Acad. Sci. USA 118:e2104592118. doi: 10.1073/pnas.2104592118, 34083444 PMC8201913

[ref10] Cantu V. A. Salamon P. Seguritan V. Redfield J. Salamon D. Edwards R. A. . (2020). PhANNs, a fast and accurate tool and web server to classify phage structural proteins. PLoS Comput. Biol. 16:e1007845. doi: 10.1371/journal.pcbi.1007845, 33137102 PMC7660903

[ref11] Cao Q. Xiao X. Bin Y. Zhao J. Zheng C. (2024). PredPVP: a stacking model for predicting phage Virion proteins based on feature selection methods. Curr. Bioinforma. 20, 817–827. doi: 10.2174/0115748936330198240924110742

[ref12] Charoenkwan P. Kanthawong S. Schaduangrat N. Yana J. Shoombuatong W. (2020b). PVPred-SCM: improved prediction and analysis of phage Virion proteins using a scoring card method. Cells 9:353. doi: 10.3390/cells9020353, 32028709 PMC7072630

[ref13] Charoenkwan P. Nantasenamat C. Hasan M. M. Shoombuatong W. (2020a). Meta-iPVP: a sequence-based meta-predictor for improving the prediction of phage virion proteins using effective feature representation. J. Comput. Aided Mol. Des. 34, 1105–1116. doi: 10.1007/s10822-020-00323-z, 32557165

[ref14] Chen T. (2016). XGBoost: A Scalable Tree Boosting System. Ithaca, NY, USA: Cornell University.

[ref15] Chen Y. Gao L. Zhang T. (2023). Stack-VTP: prediction of vesicle transport proteins based on stacked ensemble classifier and evolutionary information. BMC Bioinform. 24:137. doi: 10.1186/s12859-023-05257-5, 37029385 PMC10080812

[ref16] Chen Z. Zhao P. Li F. Leier A. Marquez-Lago T. T. Wang Y. . (2018). iFeature: a Python package and web server for features extraction and selection from protein and peptide sequences. Bioinformatics 34, 2499–2502. doi: 10.1093/bioinformatics/bty140, 29528364 PMC6658705

[ref17] Chou K. C. Shen H. B. (2007). MemType-2L: a web server for predicting membrane proteins and their types by incorporating evolution information through Pse-PSSM. Biochem. Biophys. Res. Commun. 360, 339–345. doi: 10.1016/j.bbrc.2007.06.027 17586467

[ref18] Chu Y. Guo S. Cui D. Fu X. Ma Y. (2022). DeephageTP: a convolutional neural network framework for identifying phage-specific proteins from metagenomic sequencing data. PeerJ 10:e13404. doi: 10.7717/peerj.13404, 35698617 PMC9188312

[ref19] Ding H. Feng P. M. Chen W. Lin H. (2014). Identification of bacteriophage virion proteins by the ANOVA feature selection and analysis. Mol. BioSyst. 10, 2229–2235. doi: 10.1039/c4mb00316k 24931825

[ref20] Dion M. B. Oechslin F. Moineau S. (2020). Phage diversity, genomics and phylogeny. Nat. Rev. Microbiol. 18, 125–138. doi: 10.1038/s41579-019-0311-5, 32015529

[ref21] Fang Z. Zhou H. (2021). Virionfinder: identification of complete and partial prokaryote virus virion protein from virome data using the sequence and biochemical properties of amino acids. Front. Microbiol. 12:615711. doi: 10.3389/fmicb.2021.615711, 33613485 PMC7894196

[ref22] Federici S. Nobs S. P. Elinav E. (2021). Phages and their potential to modulate the microbiome and immunity. Cell. Mol. Immunol. 18, 889–904. doi: 10.1038/s41423-020-00532-4, 32901128 PMC8115240

[ref23] Feng P. M. Ding H. Chen W. Lin H. (2013). Naïve bayes classifier with feature selection to identify phage virion proteins. Comput. Math. Methods Med. 2013:530696. doi: 10.1155/2013/530696, 23762187 PMC3671239

[ref24] Fu L. M. Niu B. F. Zhu Z. W. Wu S. T. Li W. Z. (2012). CD-HIT: accelerated for clustering the next-generation sequencing data. Bioinformatics 28, 3150–3152. doi: 10.1093/bioinformatics/bts565, 23060610 PMC3516142

[ref25] Geurts P. Ernst D. Wehenkel L. (2006). Extremely randomized trees. Mach. Learn. 63, 3–42. doi: 10.1007/s10994-006-6226-1

[ref26] Guo J. R. Bolduc B. Zayed A. A. Varsani A. Dominguez-Huerta G. Delmont T. O. . (2021). Virsorter2: a multi-classifier, expert-guided approach to detect diverse DNA and RNA viruses. Microbiome 9:37. doi: 10.1186/s40168-020-00990-y, 33522966 PMC7852108

[ref27] Han H. Zhu W. Ding C. Liu T. (2021). iPVP-MCV: a multi-classifier voting model for the accurate identification of phage virion proteins. Symmetry 13:1506. doi: 10.3390/sym13081506

[ref28] Hancock J. T. Khoshgoftaar T. M. (2020). Catboost for big data: an interdisciplinary review. J. Big Data 7:94. doi: 10.1186/s40537-020-00369-8, 33169094 PMC7610170

[ref29] Henseler J. Ringle C. M. Sarstedt M. J. (2015). A new criterion for assessing discriminant validity in variance-based structural equation modeling. J. Acad. Mark. Sci. 43, 115–135. doi: 10.1007/s11747-014-0403-8

[ref30] Jiang M. Zhao B. Luo S. Wang Q. Chu Y. Chen T. . (2021). Neuropred-fuse: an interpretable stacking model for prediction of neuropeptides by fusing sequence information and feature selection methods. Brief. Bioinform. 22:bbab310. doi: 10.1093/bib/bbab310 34396388

[ref31] Kabir M. Nantasenamat C. Kanthawong S. Charoenkwan P. Shoombuatong W. (2022). Large-scale comparative review and assessment of computational methods for phage virion proteins identification. EXCLI J. 21, 11–29. doi: 10.17179/excli2021-4411, 35145365 PMC8822302

[ref32] Ke G. Meng Q. Finley T. Wang T. Chen W. Ma W. . (2017). Lightgbm: a highly efficient gradient boosting decision tree. Adv. Neural Inf. Process. Syst. 30, 1–9. doi: 10.5555/3294996.3295074

[ref33] Li J. (2024). Area under the ROC curve has the most consistent evaluation for binary classification. PLoS One 19:e0316019. doi: 10.1371/journal.pone.0316019, 39715186 PMC11666033

[ref34] Li D. Wang Y. Hu W. Chen F. Zhao J. Chen X. . (2021). Application of machine learning classifier to *Candida auris* drug resistance analysis. Front. Cell. Infect. Microbiol. 11:742062. doi: 10.3389/fcimb.2021.742062, 34722336 PMC8554202

[ref35] Liu T. Zheng X. Wang J. (2010). Prediction of protein structural class for low-similarity sequences using support vector machine and PSI-BLAST profile. Biochimie 92, 1330–1334. doi: 10.1016/j.biochi.2010.06.013, 20600567

[ref36] Majumdar R. Karthikeyan H. Senthilnathan V. Sugumar S. (2022). Review on *Stenotrophomonas maltophilia*: an emerging multidrug- resistant opportunistic pathogen. Recent Pat. Biotechnol. 16, 329–354. doi: 10.2174/1872208316666220512121205 35549857

[ref37] Manavalan B. Shin T. H. Lee G. (2018). PVP-SVM: sequence-based prediction of phage virion proteins using a support vector machine. Front. Microbiol. 9:476. doi: 10.3389/fmicb.2018.00476, 29616000 PMC5864850

[ref38] Meng C. Zhang J. Ye X. Guo F. Zou Q. (2020). Review and comparative analysis of machine learning-based phage virion protein identification methods. Biochim. Biophys. Acta - Proteins Proteomics 1868:140406. doi: 10.1016/j.bbapap.2020.140406 32135196

[ref39] Monteiro R. Pires D. P. Costa A. R. Azeredo J. (2019). Phage therapy: going temperate? Trends Microbiol. 27, 368–378. doi: 10.1016/j.tim.2018.10.008 30466900

[ref40] Naimi A. I. Balzer L. B. (2018). Stacked generalization: an introduction to super learning. Eur. J. Epidemiol. 33, 459–464. doi: 10.1007/s10654-018-0390-z, 29637384 PMC6089257

[ref41] Noble W. (2006). What is a support vector machine? Nat. Biotechnol. 24, 1565–1567. doi: 10.1038/nbt1206-1565 17160063

[ref42] Pan Y. Gao H. Lin H. Liu Z. Tang L. Li S. (2018). Identification of bacteriophage virion proteins using multinomial naïve Bayes with g-gap feature tree. Int. J. Mol. Sci. 19:1779. doi: 10.3390/ijms19061779, 29914091 PMC6032154

[ref43] Research at UC (2019). UniProt: a worldwide hub of protein knowledge. Nucleic Acids Res. 47, D506–D515. doi: 10.1093/nar/gky1049, 30395287 PMC6323992

[ref44] Roux S. Enault F. Hurwitz B. L. Sullivan M. B. (2015). VirSorter: mining viral signal from microbial genomic data. PeerJ 3:e985. doi: 10.7717/peerj.985, 26038737 PMC4451026

[ref45] Seguritan V. Alves N. Jr. Arnoult M. Raymond A. Lorimer D. Burgin A. B. Jr. . (2012). Artificial neural networks trained to detect viral and phage structural proteins. PLoS Comput. Biol. 8:e1002657. doi: 10.1371/journal.pcbi.1002657, 22927809 PMC3426561

[ref46] Song Q. Jiang H. Liu J. (2017). Feature selection based on FDA and F-score for multi-class classification. Expert Syst. Appl. 81, 22–27. doi: 10.1016/j.eswa.2017.02.049

[ref47] Song Y. S. Liang J. Y. Lu J. Zhao X. W. (2017). An efficient instance selection algorithm for k nearest neighbor regression. Neurocomputing 251, 26–34. doi: 10.1016/j.neucom.2017.04.018

[ref48] Song Y. Y. Lu Y. (2015). Decision tree methods: applications for classification and prediction. Shanghai Arch. Psychiatry 27, 130–135. doi: 10.11919/j.issn.1002-0829.215044, 26120265 PMC4466856

[ref49] Tan J. X. Dao F. Y. Lv H. Feng P. M. Ding H. (2018). Identifying phage virion proteins by using two-step feature selection methods. Molecules 23:2000. doi: 10.3390/molecules23082000, 30103458 PMC6222849

[ref50] Tang J. Deng C. Huang G. B. (2015). Extreme learning machine for multilayer perceptron. IEEE Trans. Neural Netw. Learn. Syst. 27, 809–821. doi: 10.1109/TNNLS.2015.2424995, 25966483

[ref51] Thung T. Y. White M. E. Dai W. Wilksch J. J. Bamert R. S. Rocker A. . (2021). Component parts of bacteriophage Virions accurately defined by a machine-learning approach built on evolutionary features. mSystems 6:e0024221. doi: 10.1128/mSystems.00242-21, 34042467 PMC8269216

[ref52] van Smeden M. Moons K. G. M. de Groot J. A. H. Collins G. S. Altman D. G. Eijkemans M. J. C. . (2019). Sample size for binary logistic prediction models: beyond events per variable criteria. Stat. Methods Med. Res. 28, 2455–2474. doi: 10.1177/0962280218784726, 29966490 PMC6710621

[ref53] Wang R. (2012). AdaBoost for feature selection, classification and its relation with SVM, a review. Phys. Procedia 25, 800–807. doi: 10.1016/j.phpro.2012.03.160

[ref54] Wang J. Yang B. Revote J. Leier A. Marquez-Lago T. T. Webb G. . (2017). POSSUM: a bioinformatics toolkit for generating numerical sequence feature descriptors based on PSSM profiles. Bioinformatics 33, 2756–2758. doi: 10.1093/bioinformatics/btx302 28903538

[ref55] Warwick-Dugdale J. Buchholz H. H. Allen M. J. Temperton B. (2019). Host-hijacking and planktonic piracy: how phages command the microbial high seas. Virol. J. 16:15. doi: 10.1186/s12985-019-1120-1, 30709355 PMC6359870

[ref56] Xiong Y. Wang Q. Yang J. Zhu X. Wei D. Q. (2018). PredT4SE-stack: prediction of bacterial type IV secreted effectors from protein sequences using a stacked ensemble method. Front. Microbiol. 9:2571. doi: 10.3389/fmicb.2018.02571, 30416498 PMC6212463

[ref57] Yang R. Liu J. Zhang L. (2023). ECAmyloid: an amyloid predictor based on ensemble learning and comprehensive sequence-derived features. Comput. Biol. Chem. 104:107853. doi: 10.1016/j.compbiolchem.2023.107853 36990028

[ref58] Zhang Q. Liu P. Wang X. Zhang Y. Han Y. Yu B. (2021). StackPDB: predicting DNA-binding proteins based on XGB-RFE feature optimization and stacked ensemble classifier. Appl. Soft Comput. 99:106921. doi: 10.1016/j.asoc.2020.106921

[ref59] Zhang L. Zhang C. Gao R. Yang R. (2015). An ensemble method to distinguish bacteriophage Virion from non-Virion proteins based on protein sequence characteristics. Int. J. Mol. Sci. 16, 21734–21758. doi: 10.3390/ijms160921734, 26370987 PMC4613277

[ref60] Zhang C. Zhang Y. Shi X. Almpanidis G. Fan G. Shen X. (2019). On incremental learning for gradient boosting decision trees. Neural. Process. Lett. 50, 957–987. doi: 10.1007/s11063-019-09999-3

[ref61] Zou L. Nan C. Hu F. (2013). Accurate prediction of bacterial type IV secreted effectors using amino acid composition and PSSM profiles. Bioinformatics 29, 3135–3142. doi: 10.1093/bioinformatics/btt554, 24064423 PMC5994942

