# Supplementary material for: StackPVP: a stacked ensemble classification framework for predicting phage virion proteins using integrated evolutionary features
Source: Front Microbiol. 2026 Mar 18;17:1729937. doi: 10.3389/fmicb.2026.1729937 (PMC13038940; doi:10.3389/fmicb.2026.1729937)
Supplement: Supplementary file 1 [file Table_1.docx]

Table S1. Performance of base classifier models using sequence and evolutionary features on the test dataset.

| **All features** |  | **ACC** | **Sn** | **Sp** | **Precision** | **F1** | **MCC** | **AUC** |
| --- | --- | --- | --- | --- | --- | --- | --- | --- |
| AA_Comp | KNN | 0.7143 | 0.8413 | 0.5873 | 0.6709 | 0.7465 | 0.4431 | 0.8185 |
|  | RF | 0.8175 | 0.7460 | 0.8889 | 0.8704 | 0.8034 | 0.6415 | 0.8738 |
|  | XGBoost | 0.8175 | 0.7302 | 0.9048 | 0.8846 | 0.8000 | 0.6448 | 0.8718 |
|  | GBDT | 0.8175 | 0.7778 | 0.8571 | 0.8448 | 0.8099 | 0.6369 | 0.8707 |
|  | EF | 0.7857 | 0.6825 | 0.8889 | 0.8600 | 0.7611 | 0.5840 | 0.8690 |
|  | LightGBM | 0.7937 | 0.7302 | 0.8571 | 0.8364 | 0.7797 | 0.5921 | 0.8662 |
|  | ANN | 0.8016 | 0.7143 | 0.8889 | 0.8654 | 0.7826 | 0.6126 | 0.8808 |
|  | SVM | 0.8254 | 0.7937 | 0.8571 | 0.8475 | 0.8197 | 0.6521 | 0.9098 |
|  | DT | 0.7460 | 0.8095 | 0.6825 | 0.7183 | 0.7612 | 0.4961 | 0.7444 |
|  | MLP | 0.7698 | 0.7778 | 0.7619 | 0.7656 | 0.7717 | 0.5398 | 0.8408 |
|  | AdaBoost | 0.7857 | 0.7619 | 0.8095 | 0.8000 | 0.7805 | 0.5721 | 0.8535 |
|  | CatBoost | 0.8095 | 0.7778 | 0.8413 | 0.8305 | 0.8033 | 0.6203 | 0.8806 |
| PSSM | KNN | 0.7222 | 0.8571 | 0.5873 | 0.6750 | 0.7552 | 0.4616 | 0.8199 |
|  | RF | 0.8651 | 0.8254 | 0.9048 | 0.8966 | 0.8595 | 0.7325 | 0.8995 |
|  | XGBoost | 0.8571 | 0.8413 | 0.8730 | 0.8689 | 0.8548 | 0.7146 | 0.9093 |
|  | GBDT | 0.8492 | 0.8095 | 0.8889 | 0.8793 | 0.8430 | 0.7006 | 0.8954 |
|  | EF | 0.8651 | 0.8254 | 0.9048 | 0.8966 | 0.8595 | 0.7325 | 0.8755 |
|  | LightGBM | 0.8492 | 0.8413 | 0.8571 | 0.8548 | 0.8480 | 0.6985 | 0.9058 |
|  | ANN | 0.8492 | 0.8730 | 0.8254 | 0.8333 | 0.8527 | 0.6992 | 0.9244 |
|  | SVM | 0.8810 | 0.9206 | 0.8413 | 0.8529 | 0.8855 | 0.7643 | 0.9264 |
|  | DT | 0.7063 | 0.6349 | 0.7778 | 0.7407 | 0.6838 | 0.4170 | 0.6807 |
|  | MLP | 0.8413 | 0.8095 | 0.8730 | 0.8644 | 0.8361 | 0.6839 | 0.8944 |
|  | AdaBoost | 0.8016 | 0.7619 | 0.8413 | 0.8276 | 0.7934 | 0.6051 | 0.8687 |
|  | CatBoost | 0.8333 | 0.8254 | 0.8413 | 0.8387 | 0.8320 | 0.6668 | 0.9035 |

Table S2. Performance of base classifier models using various dimensionality reduction methods on the test dataset.

| **PSSM** |  | **ACC** | **Sn** | **Sp** | **Precision** | **F1** | **MCC** | **AUC** |
| --- | --- | --- | --- | --- | --- | --- | --- | --- |
| F-score | KNN | 0.7540 | 0.8571 | 0.6508 | 0.7105 | 0.7770 | 0.5191 | 0.8345 |
|  | RF | 0.8413 | 0.7937 | 0.8889 | 0.8772 | 0.8333 | 0.6857 | 0.8997 |
|  | XGBoost | 0.8492 | 0.8254 | 0.8730 | 0.8667 | 0.8455 | 0.6992 | 0.9131 |
|  | GBDT | 0.8492 | 0.8254 | 0.8730 | 0.8667 | 0.8455 | 0.6992 | 0.8927 |
|  | EF | 0.8413 | 0.7778 | 0.9048 | 0.8909 | 0.8305 | 0.6881 | 0.8662 |
|  | LightGBM | 0.8571 | 0.8254 | 0.8889 | 0.8814 | 0.8525 | 0.7157 | 0.9007 |
|  | ANN | 0.8413 | 0.8571 | 0.8254 | 0.8308 | 0.8438 | 0.6829 | 0.9131 |
|  | SVM | 0.8730 | 0.9048 | 0.8413 | 0.8507 | 0.8769 | 0.7475 | 0.9237 |
|  | DT | 0.7540 | 0.7302 | 0.7778 | 0.7667 | 0.7480 | 0.5085 | 0.6980 |
|  | MLP | 0.7937 | 0.7619 | 0.8254 | 0.8136 | 0.7869 | 0.5885 | 0.8667 |
|  | AdaBoost | 0.8175 | 0.7619 | 0.8730 | 0.8571 | 0.8067 | 0.6389 | 0.8763 |
|  | CatBoost | 0.8492 | 0.8413 | 0.8571 | 0.8548 | 0.8480 | 0.6985 | 0.9136 |
| Var | KNN | 0.7619 | 0.8571 | 0.6667 | 0.7200 | 0.7826 | 0.5336 | 0.8454 |
|  | RF | 0.8571 | 0.8095 | 0.9048 | 0.8947 | 0.8500 | 0.7175 | 0.8901 |
|  | XGBoost | 0.8651 | 0.8254 | 0.9048 | 0.8966 | 0.8595 | 0.7325 | 0.9048 |
|  | GBDT | 0.8651 | 0.8254 | 0.9048 | 0.8966 | 0.8595 | 0.7325 | 0.8957 |
|  | EF | 0.8651 | 0.8413 | 0.8889 | 0.8833 | 0.8618 | 0.7310 | 0.8743 |
|  | LightGBM | 0.8492 | 0.8254 | 0.8730 | 0.8667 | 0.8455 | 0.6992 | 0.9043 |
|  | ANN | 0.8016 | 0.8571 | 0.7460 | 0.7714 | 0.8120 | 0.6069 | 0.8705 |
|  | SVM | 0.8810 | 0.9206 | 0.8413 | 0.8529 | 0.8855 | 0.7643 | 0.9085 |
|  | DT | 0.7302 | 0.7302 | 0.7302 | 0.7302 | 0.7302 | 0.4603 | 0.7274 |
|  | MLP | 0.8333 | 0.7937 | 0.8730 | 0.8621 | 0.8264 | 0.6688 | 0.8864 |
|  | AdaBoost | 0.8016 | 0.7619 | 0.8413 | 0.8276 | 0.7934 | 0.6051 | 0.8702 |
|  | CatBoost | 0.8571 | 0.8571 | 0.8571 | 0.8571 | 0.8571 | 0.7143 | 0.8995 |
| RFECV | KNN | 0.7460 | 0.8571 | 0.6349 | 0.7013 | 0.7714 | 0.5047 | 0.8381 |
|  | RF | 0.8492 | 0.7937 | 0.9048 | 0.8929 | 0.8403 | 0.7028 | 0.8997 |
|  | XGBoost | 0.8571 | 0.8413 | 0.8730 | 0.8689 | 0.8548 | 0.7146 | 0.9148 |
|  | GBDT | 0.8651 | 0.8254 | 0.9048 | 0.8966 | 0.8595 | 0.7325 | 0.8992 |
|  | EF | 0.8571 | 0.8095 | 0.9048 | 0.8947 | 0.8500 | 0.7175 | 0.8776 |
|  | LightGBM | 0.8413 | 0.8254 | 0.8571 | 0.8525 | 0.8387 | 0.6829 | 0.9098 |
|  | ANN | 0.8492 | 0.8413 | 0.8571 | 0.8548 | 0.8480 | 0.6985 | 0.9229 |
|  | SVM | 0.8968 | 0.9206 | 0.8730 | 0.8788 | 0.8992 | 0.7946 | 0.9307 |
|  | DT | 0.7381 | 0.7302 | 0.7460 | 0.7419 | 0.7360 | 0.4763 | 0.6969 |
|  | MLP | 0.8175 | 0.7778 | 0.8571 | 0.8448 | 0.8099 | 0.6369 | 0.9002 |
|  | AdaBoost | 0.8095 | 0.7619 | 0.8571 | 0.8421 | 0.8000 | 0.6219 | 0.8702 |
|  | CatBoost | 0.8410 | 0.8410 | 0.8410 | 0.8410 | 0.8410 | 0.6830 | 0.9090 |

Table S3. Cross-validation results for different meta-classifier models on training dataset.

| **meta-classifier** | **Dataset** | **ACC** | **Sn** | **Sp** | **Precision** | **F1** | **MCC** | **AUC** |
| --- | --- | --- | --- | --- | --- | --- | --- | --- |
| LR | F | 0.9975 | 0.9943 | 1.0000 | 1.0000 | 0.9971 | 0.9950 | 0.9999 |
|  | Var | 0.9975 | 0.9943 | 1.0000 | 1.0000 | 0.9971 | 0.9950 | 0.9999 |
|  | RFECV | 0.9975 | 0.9943 | 1.0000 | 1.0000 | 0.9971 | 0.9950 | 0.9999 |
|  | F_Var | 0.9975 | 0.9943 | 1.0000 | 1.0000 | 0.9971 | 0.9950 | 0.9998 |
|  | F_RFECV | 0.9975 | 0.9943 | 1.0000 | 1.0000 | 0.9971 | 0.9950 | 0.9998 |
|  | Var_RFECV | 0.9975 | 0.9943 | 1.0000 | 1.0000 | 0.9971 | 0.9950 | 0.9998 |
|  | All_three | 0.9975 | 0.9943 | 1.0000 | 1.0000 | 0.9971 | 0.9950 | 0.9998 |
| RF | F | 0.9542 | 0.9513 | 0.9563 | 0.9432 | 0.9472 | 0.9067 | 0.9924 |
|  | Var | 0.9653 | 0.9656 | 0.9651 | 0.9547 | 0.9601 | 0.9295 | 0.9958 |
|  | RFECV | 0.9529 | 0.9771 | 0.9345 | 0.9191 | 0.9472 | 0.9062 | 0.9956 |
|  | F_Var | 0.9542 | 0.9599 | 0.9498 | 0.9358 | 0.9477 | 0.9071 | 0.9942 |
|  | F_RFECV | 0.9306 | 0.9713 | 0.8996 | 0.8805 | 0.9237 | 0.8638 | 0.9941 |
|  | Var_RFECV | 0.9690 | 0.9742 | 0.9651 | 0.9551 | 0.9645 | 0.9372 | 0.9955 |
|  | All_three | 0.9467 | 0.9656 | 0.9323 | 0.9158 | 0.9400 | 0.8932 | 0.9932 |
| SVM | F | 0.9864 | 0.9943 | 0.9803 | 0.9747 | 0.9844 | 0.9724 | 0.9994 |
|  | Var | 0.9975 | 0.9943 | 1.0000 | 1.0000 | 0.9971 | 0.9950 | 0.9994 |
|  | RFECV | 0.9827 | 0.9943 | 0.9738 | 0.9666 | 0.9802 | 0.9651 | 0.9991 |
|  | F_Var | 0.9864 | 0.9943 | 0.9803 | 0.9747 | 0.9844 | 0.9724 | 0.9956 |
|  | F_RFECV | 0.9827 | 0.9943 | 0.9738 | 0.9666 | 0.9802 | 0.9651 | 0.9966 |
|  | Var_RFECV | 0.9901 | 0.9943 | 0.9869 | 0.983 | 0.9886 | 0.9799 | 0.9985 |
|  | All_three | 0.9851 | 0.9943 | 0.9782 | 0.9720 | 0.9830 | 0.9700 | 0.9946 |
